# Inflammatory Bowel Disease: Understanding Therapeutic Effects of Distinct Molecular Inhibitors as the Key to Current and Future Advanced Therapeutic Strategies

**DOI:** 10.3390/biomedicines13112667

**Published:** 2025-10-30

**Authors:** Alice Laffusa, Cesare Burti, Chiara Viganò, Francesca Poggi, Laura Grieco, Vincenzo Occhipinti, Salvatore Greco, Stefania Orlando

**Affiliations:** 1 Gastroenterology and Endoscopy Unit, Papa Giovanni XXIII Hospital, 24127 Bergamo, Italy; 2 Gastroenterology, Fondazione IRCCS San Gerardo dei Tintori, 20900 Monza, Italy; 3 Division of Gastroenterology, Papa Giovanni XXIII Hospital, 24127 Bergamo, Italy; 4 Department of Medicine and Surgery, University of Milano Bicocca, 20126 Milan, Italy

**Keywords:** inflammatory bowel disease, biologics, small molecules, combination therapy, extraintestinal manifestations

## Abstract

The pathogenesis of Inflammatory Bowel Disease is complex and not completely understood, resulting from multifactorial interactions between genetic predisposition, environmental triggers, and dysregulation of both innate and adaptive immune responses. Cytokines, produced by dysregulated immune cells, trigger chronic intestinal inflammation leading to tissue damage, carcinogenesis, and disease perpetuation. Current advanced therapies—including tumor necrosis factor (TNF)-α antagonists, adhesion and trafficking inhibitors (such as anti-integrin agents and sphingosine-1-phosphate receptor modulators), interleukin inhibitors, and Janus kinase inhibitors—have improved patient outcomes, but targeting a single inflammatory pathway is often insufficient for long-term disease control. To further improve therapeutic efficacy, novel approaches are under investigation, including advanced combination therapies that simultaneously inhibit multiple pro-inflammatory pathways and microbiome-based treatments to restore intestinal homeostasis. In this evolving therapeutic scenario, precision medicine and advanced combination therapies appear promising for breaking through the current therapeutic ceiling. This review highlights current knowledge on the role of cytokines in IBD pathogenesis and explores how their modulation can modify and control disease course.

## 1. Introduction

Inflammatory Bowel Disease (IBD), including Ulcerative Colitis (UC) and Crohn’s disease (CD), comprises chronic immune-mediated disorders of the gastrointestinal tract. Although their pathogenesis is not fully understood, genetic predisposition, environmental factors and intestinal barrier disfunction contribute to the immune dysregulation that drives disease onset [1]. The intestinal barrier separates the lamina propria and deeper tissues from the intestinal lumen, and its damage is considered both a cause and a consequence of IBD. A crucial component of this barrier is the gut microbiota, which co-evolves with the host and contributes to epithelial integrity, immune regulation, metabolic balance, and nutrient provision. Imbalances between beneficial and harmful bacteria, as well as reduced biodiversity and species richness, can disrupt mucosal homeostasis and promote inflammation. Innate immune cells, particularly macrophages, mediate responses to microbial products through pattern recognition receptors (PRRs). Under homeostatic conditions, prolonged exposure to microbial signals enhances antimicrobial defences while limiting inflammatory cytokines. Conversely, PRR signalling defects lead to increased bacterial burden, dysbiosis, excessive inflammatory mediator production, immune dysregulation, and intestinal inflammation. Dysregulation of this gut–immune–microbiome axis, commonly termed “leaky gut,” exacerbate IBD by perpetuating mucosal damage and chronic inflammation [1,2,3] through multiple cytokine-dependent pathways, with a significant interindividual heterogeneity.

Understanding the alterations in the intestinal cytokine network is central to IBD management, as pharmacological treatment is primarily aimed at suppressing the inappropriate immune response. Historically, non-specific immunosuppressive agents such as corticosteroids, thiopurines, and other immunomodulators were widely used [4]. Today, these drugs are mainly reserved for induction of remission in acute flares or in combination with advanced therapies.

Over the past decades, treatment options for IBD have expanded considerably. Five major classes of advanced therapies, comprising biologics and oral small molecules, are currently approved, each targeting different points of the inflammatory cascade: tumor necrosis factor (TNF)-α antagonists, adhesion and trafficking inhibitors (including anti-integrin agents and sphingosine1-phosphate receptor modulators), interleukin Inhibitors, and Janus kinase inhibitors. An overview of representative clinical trials and their outcomes is provided in Table 1 for UC and in Table 2 for CD. Despite these advances, up to one-third of patients show primary non-response to initial therapy, and approximately half lose response over time [5].

Ongoing research is therefore focused on identifying novel therapeutic targets and evaluating combination strategies to break through the therapeutic ceiling. In this review, we discuss the key molecular mechanisms underlying IBD and the pharmacological strategies derived from their inhibition or modulation, with particular attention to emerging approaches aimed at targeting patients with extraintestinal manifestations and those who fail to respond to currently available therapies.

## 2. Tumor Necrosis Factor (TNF)-α Antagonists

**Figure 1:**
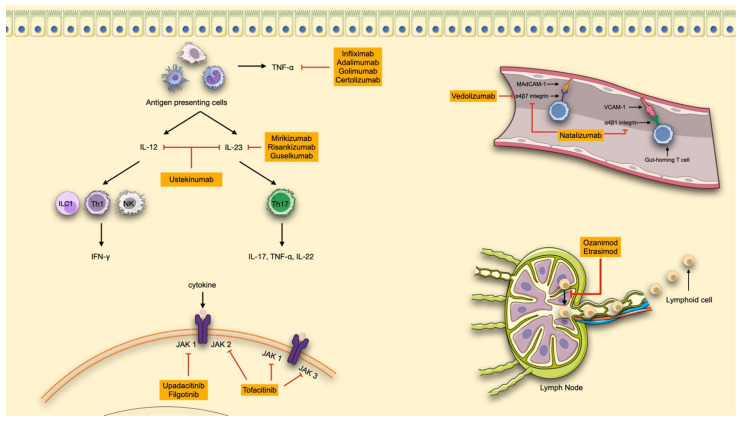
Advanced therapeutic strategies in IBD. Available therapies are shown in orange boxes. TNF, tumor necrosis factor; IFN, interferon; IL, interleukin; ILC, innate lymphoid cell; Th, T helper; NK, natural killer cells; MAdCAM-1, mucosal addressin cell adhesion molecule 1; VCAM-1, vascular cell adhesion molecule-1; JAK, Janus kinase. The red lines connect the molecules to their target of action. (made with NIH Bioart, Smart Servier Medical Art and Bioicons).

Tumor necrosis factor alpha (TNF-α) is a cytokine primarily produced by immune cells, particularly macrophages and monocytes, which plays a central role in the dysregulated immune response underlying chronic inflammation in IBD. TNF-α exists in two biologically active forms: a transmembrane form (mTNF), which is involved in tissue repair processes, and a soluble form (sTNF), which is widely expressed and responsible for promoting inflammation [38,39,40]. sTNF exerts its effects by activating several pro-inflammatory pathways—such as MAPK, NF-κB, and the caspase cascade—ultimately leading to cytokine and chemokine production, chronic tissue damage, and fibrosis [40]. The advent of anti-TNF-α therapies has revolutionized IBD management by neutralizing this cytokine’s effects, reducing the infiltration of inflammatory immune cells, and promoting mucosal healing [39,40] (Figure 1).

Infliximab (IFX), a chimeric monoclonal antibody targeting TNF-α and administered intravenously (IV), was the first biologic agent approved for IBD treatment. In the phase 3 ACCENT-1 trial IFX demonstrated efficacy in maintaining clinical remission in patients with moderate-to-severe CD, with 39% of patients in remission at week 30 [22], and in achieving endoscopic remission in approximately 50% of patients by week 54. Additionally, the ACCENT-2 trial confirmed IFX efficacy in fistulizing CD, with 36% of patients achieving fistula closure at week 54 compared with 19% in the placebo group [41].

IFX has also proven effective in UC. The ACT 1 and ACT 2 trials showed that over 65% of patients with refractory UC achieved a clinical response at week 8 with IFX, compared with 33% with placebo, and experienced significant endoscopic improvement. Moreover, approximately 60% of patients achieved mucosal healing during both induction and maintenance therapy [6].

The CYSIF trial compared IFX versus cyclosporine in patients with steroid-refractory acute severe UC (ASUC). Treatment failure in this specific condition occurred in 60% of patients receiving cyclosporine and 54% receiving IFX, with no statistically significant difference between the two groups [42] (Table 3).

To address the loss of response due to the development of anti-drug antibodies, combination therapy with IFX and immunomodulators (IMM) has been explored. The SONIC trial showed that IFX combined with azathioprine was more effective than either agent alone in inducing clinical remission, achieving mucosal healing, and limiting the development of anti-IFX antibodies in patients with moderate-to-severe CD [23].

More recently, a subcutaneous formulation of an IFX biosimilar has been approved. The LIBERTY-CD and LIBERTY-UC trials, together with the REMSWITCH study, demonstrated the non-inferiority of the subcutaneous formulation compared with intravenous IFX in maintaining clinical remission. This formulation appears to reduce the risk of immunogenicity and offers greater convenience for the patients, by avoiding clinic visits thus potentially increasing adherence [45,46].

The CONNECT IBD, a prospective cohort European observational study, involved 2543 patients with both UC and CD and demonstrated that IFX (both originator and biosimilar) is effective in the real world setting for maintaining remission [47].

Adalimumab, a fully human anti-TNF-α antibody, is the second anti-TNFα agent approved for IBD. It is administered subcutaneously (SC) and has demonstrated strong efficacy in both induction and maintenance of remission in moderate-to-severe CD and UC.

The CLASSIC-I induction trial, which included anti-TNFα-naive patients with CD, showed a significantly higher clinical remission rate at week 4 with adalimumab compared with placebo (36% vs. 12%) [24]. The CLASSIC-II maintenance trial confirmed the efficacy of Adalimumab in maintaining CD remission at week 56, with rates of 79% and 83% for patients receiving 40 mg every other week and weekly, respectively, compared with 44% with placebo [25]. In CD patients previously exposed to IFX, the GAIN trial showed that adalimumab remained effective, with 21% of patients achieving clinical remission at week 4 compared with 7% in the placebo group [26]. The EXTEND trial further demonstrated that adalimumab is also effective in achieving mucosal healing, with 24% of patients reaching this endpoint at week 52, versus 0% in the placebo group [27]. The DIAMOND trial evaluated adalimumab in combination with IMM versus adalimumab monotherapy in patients with CD naïve to both agents. Although no significant differences were found in clinical remission or maintenance rates, combination therapy resulted in higher rates of mucosal healing at week 26 (84% vs. 63%) and showed a trend toward reduced immunogenicity [48]. However, current international guidelines recommend combination therapy primarily for IFX, given the stronger supporting evidence [49,50]. More recently, the SEAUVE trial, a head-to-head randomized controlled trial, compared adalimumab to ustekinumab (an anti-interleukin-12/23 agent) in biologic-naïve patients with moderate-to-severe CD. The results demonstrated non-inferiority of ustekinumab compared with adalimumab in both clinical (65% vs. 61%) and endoscopic remission (36% vs. 35%) at week 52 [44] (Table 3).

In the ULTRA-I trial, 18% of patients with moderate-to-severe UC achieved clinical remission at week 8 with adalimumab, versus 9% with placebo [7]. The ULTRA-II study confirmed the efficacy of adalimumab as maintenance therapy in UC, with 17% of patients in clinical remission at week 52 compared with 8% in the placebo group [8].

Golimumab and Certolizumab-pegol are two additional anti-TNFα agents, though they are less commonly used in routine clinical practice compared with IFX and adalimumab. Golimumab, administered subcutaneously (SC), has been approved for the treatment of UC. The PURSUIT-SC induction trial and the subsequent PURSUIT-M maintenance trial demonstrated its efficacy in inducing clinical remission and mucosal healing at week 6, as well as maintaining remission at week 52, compared with placebo [9,10]. However, the PURSUIT-IV trial, which evaluated the IV administration of golimumab, failed to demonstrate a significant benefit in inducing remission in patients with moderate-to-severe UC [51]. Certolizumab pegol is approved for the treatment of moderate-to-severe CD. In the PRECiSE 1 trial, certolizumab was shown to be more effective than placebo in inducing a clinical response at week 4 (35% vs. 27%) [28]. The PRECiSE 2 trial, which included patients who had initially responded to induction therapy, demonstrated sustained clinical remission was achieved at week 26 in 48% of patients receiving certolizumab compared with 29% in the placebo group [29]. The PRECiSE 3 extension trial further confirmed the long-term efficacy and safety of certolizumab in this patient population [52]. Certolizumab pegol has been approved in the United States since 2008 for use in patients with moderate-to-severe CD refractory to conventional therapies. However, its availability remains limited in other regions, including Europe, where it is not currently authorized for IBD treatment.

## 3. Interleukin-Inhibitors

Interleukins 12 and 23 (IL-12 and IL-23) are two crucial pro-inflammatory cytokines involved in the immune response and in the pathogenesis of IBD. Both are produced by antigen-presenting cells (APCs), such as dendritic cells and macrophages, which present antigens to T cells to initiate the immune response [53].

IL-12 is a heterodimer, composed of the p40 subunit, shared with IL-23, and the unique p35 subunit. IL-12 plays a central role in the activation of natural killer (NK) cells, type 1 cytotoxic T-cells (Tc1), and innate lymphoid cells type 1 (ILC1). It also promotes the differentiation of naïve T-cells into T-helper 1 (Th1) cells, which secrete Interferon-gamma (INF γ), further amplifying the inflammatory cascade [4].

IL-23, also a heterodimer, shares the p40 subunit with IL-12 but pairs it with a unique p19 subunit. Once produced, IL-23 drives the differentiation, proliferation, and maintenance of T-helper 17 (Th17) cells, which secrete IL-17, IL-22 and TNF-α, cytokines implicated in sustaining inflammation and promoting tissue fibrosis. Beyond its role in Th17 activation, IL-23 exerts broader regulatory effects on both innate and adaptive immune response, being its receptor expressed on several immune cells, including intraepithelial lymphocytes, NK cells, and granulocytes [54]. Moreover, IL-23 inhibits anti-TNF-induced apoptosis in mucosal T cells and plays a crucial role in patients who do not respond to anti-TNF-α therapy, as these patients show a significant increase in mucosal levels of IL-23p19 [55].

Both IL-12 and IL-23 are essential for mucosal immunity and contribute to the maintenance of the intestinal epithelial barrier under physiological conditions. The first therapeutic efforts targeted the shared p40 subunit, leading to the development of ustekinumab, which inhibits both IL-12 and IL-23 [53]. However, dysregulation of IL-23 has been shown to play a particularly prominent role in IBD pathogenesis. Elevated levels of IL-23 and Th17 cells have been found in intestinal tissues of IBD patients, suggesting a greater pro-inflammatory impact compared with IL-12. The understanding of the central role of IL-23 in peripheral tissue inflammation has promoted the development of more selective anti-IL-23 therapies targeting specifically the p19 subunit. These agents, mirikizumab, risankizumab and guselkumab, have shown promising results and offer a more targeted approach to modulating chronic intestinal inflammation [4] (Figure 1). IL-inhibitors currently available for IBD treatment include an IV induction phase, followed by SC maintenance therapy.

Ustekinumab, the first anti-IL agent developed for IBD, targets both IL-12 and IL-23 by selectively binding to their shared p40 subunit. Ustekinumab was initially approved for moderate-to-severe CD, based on the UNITI trial [30], which demonstrated significantly higher clinical response rates compared with placebo (34% vs. 6.5%). In biologic-naïve patients with CD, the phase-3 head-to-head SEAVUE trial compared ustekinumab with adalimumab, showing that both agents were highly effective, with no significant differences in clinical remission or endoscopic response at week 52 [44]. The efficacy of ustekinumab in moderate-to-severe UC, was established in the phase 3 UNIFI trial, which demonstrated superiority over placebo in achieving clinical remission (16% vs. 5%) and endoscopic improvement [11].

Risankizumab, a selective IL-23 inhibitor, was first approved in 2022 for moderate-to-severe CD. In the ADVANCE induction trial [31], conducted on CD patients with prior failure of conventional or biologic therapies, risankizumab achieved higher rates of clinical remission (45% vs. 25%, *p* < 0.001) and endoscopic response (40% vs. 12%, *p* < 0.0001) compared with placebo. Clinical remission rates were consistent regardless of prior biologic exposure, while endoscopic responses were higher in biologic-naïve patients. In the MOTIVATE induction trial [31], limited to patients with prior biologic-failure, risankizumab still outperformed placebo in both clinical remission (42% vs. 19%, *p* < 0.0001) and endoscopic response (29% vs. 11%, *p* < 0.0001). In the FORTIFY maintenance trial [32], risankizumab maintained superiority over placebo at week 52 for both endpoints, independently of prior biologic exposure. In a recent head-to-head trial in CD patients with unacceptable side effects with, or inadequate response to anti-TNF- α, risankizumab was non-inferior to ustekinumab for clinical remission at week 42 and superior for endoscopic remission at week 48 [56]. For moderate-to-severe UC, the INSPIRE induction trial [12] demonstrated that risankizumab was more effective than placebo in achieving clinical remission at weeks 12 (20.3% vs. 6.2%; *p* < 0.00001). In the phase 3 COMMAND maintenance trial [13], week 52 outcomes favored risankizumab, with higher rates of clinical remission (40.2% vs. 25.1%; *p* < 0.001) and endoscopic improvement (50.8% vs. 31.7%; *p* < 0.001) compared with placebo. Since its approval for IBD, several real-world cohort studies have confirmed its effectiveness in clinical practice [53].

Mirikizumab, another selective IL-23 inhibitor, was the first approved for moderate-to-severe UC. Approval was based on the LUCENT-1 induction and LUCENT-2 maintenance trials [14], which demonstrated significant differences compared with placebo in both induction of remission (24.2% vs. 13.3%; *p* < 0.001) and long-term remission (49.9% vs. 25.1%; *p* < 0.001), also in patients refractory to biologics (including ustekinumab) or JAK inhibitors. The LUCENT-3 extension study [57] confirmed efficacy through 104 weeks of treatment. In the phase 3 VIVID-1 trial [33], which enrolled CD patients with prior failure of conventional or biologic therapy, mirikizumab achieved higher rates of clinical remission (45.4% vs. 19.6%, *p* < 0.0001) and endoscopic response (38% vs. 9%, *p* < 0.0001) compared with placebo. These results were consistent regardless of prior biologic exposure. Based on these findings, mirikizumab was approved in the United States in in January 2025 for the treatment of CD. In Europe, regulatory approval for CD is currently under review.

Guselkumab, the newest IL-23 inhibitor available for IBD treatment, was first approved for UC in 2024 in the United States, based on results from the QUASAR phase 3 induction and maintenance [15] trials. These studies enrolled patients with moderate-to-severe UC, who had experienced inadequate response, loss of response or intolerance to conventional or biologic therapies. In the induction phase guselkumab achieved clinical remission in 23% of patients versus 8% in the placebo group (*p* < 0.0001), while endoscopic response rates were 27% and 11%, respectively (*p* < 0.0001). In the maintenance phase, clinical remission rates were significantly higher among patients receiving guselkumab every 4 weeks (50%; *p* < 0.0001) or every 8 weeks (45%; *p* < 0.0001) compared with placebo (19%). Efficacy during both induction and maintenance phases was consistent regardless of prior exposure to biologics or JAK inhibitors. The ASTRO phase 3 trial, further confirmed the efficacy of guselkumab administered subcutaneously for induction, demonstrating that both IV and SC induction regimens were highly effective [58]. More recently, the GRAVITI trial [34] led to the FDA approval of Guselkumab in March 2025 for the treatment of moderate-to severe CD. This phase 3 study evaluated SC induction followed by SC maintenance and demonstrated significantly higher rates of clinical remission and endoscopic response at week 12 in patients receiving guselkumab compared with placebo, both in biologic-naïve patients and in those with inadequate response or intolerance to other biologics. Promising data are also anticipated from the ongoing phase 3 GALAXI- 2 and GALAXI-3 trials [59], which are assessing mirikizumab IV induction followed by SC maintenance, with placebo and ustekinumab as comparators, in patients with CD.

In the past few months guselkumab has also obtained regulatory approval in Europe for both UC and CD, further expanding its therapeutic availability.

## 4. Adhesion and Trafficking Inhibitors

In the chronic inflammation that characterizes IBD, immune cell trafficking—encompassing leukocyte circulation, migration, and infiltration into target organs—plays a central role. Following their activation in lymph nodes, lymphocytes involved in inflammatory response exit these sites and migrate toward target tissues, guided by a concentration gradient of sphingosine-1-phosphate (S1P), a phospholipid signalling molecule detected by S1P receptors (S1PR) [53]. S1PR are bioactive metabolites derived from essential components of the cell membrane and are located on the cell surface. They interact with five G protein-coupled receptors (S1P1 to S1P5), triggering a broad spectrum of physiological responses. Among them S1P1, S1P4, and S1P5 are predominantly expressed on leukocytes, NK cells, and dendritic cells, where they regulate lymphocyte differentiation, proliferation, migration, cytokine production, and vascular permeability—making them an important therapeutic target [4] (Figure 1).

Once reaching target organs, leukocytes infiltrate inflammatory sites through interactions between integrins, expressed on their surface, and corresponding adhesion molecules (integrin ligands) on vascular endothelial cells. In the intestinal tract specifically, lymphocytes expressing α4β7 integrin bind to mucosal addressin cell adhesion molecule-1 (MAdCAM-1), which is primarily expressed on the vascular endothelium in the gastrointestinal tract. This interaction represents another promising therapeutic target for selectively blocking lymphocyte migration to the gut [54] (Figure 1).

Both anti-integrin agents and S1PR modulators are currently available therapeutic strategies aimed at inhibiting immune cell trafficking in the treatment of IBD.

### 4.1. Anti-Integrin Agents

Vedolizumab is a humanized monoclonal antibody that selectively targets the α4β7 integrin on gut-homing lymphocytes, thereby reducing intestinal inflammation without inducing significant systemic immunosuppression. This gut-selective mechanism accounts for its favorable safety profile, with a low incidence of serious infections [60]. In the pivotal GEMINI 1 trial, vedolizumab induced a significantly higher clinical response at 6 weeks in patients with moderate-to-severe UC (47.1% vs. 25.5%,) and maintained remission at 52 weeks in a greater proportion of patients compared with placebo (41.8% vs. 15.9%) [16]. Long-term extension demonstrated sustained remission for up to 3 years in responders, with real-world data confirming these outcomes [61,62,63]. The VICTORY consortium study further reported cumulative clinical remission and endoscopic remission rates of 51% and 41%, respectively, at 1 year in anti TNF-experienced patients [64]. The VARSITY head-to-head trial compared vedolizumab to adalimumab in moderate-to-severe UC, showing superior efficacy of vedolizumab in achieving 1-year clinical remission (31.3% vs. 22.5%) and endoscopic improvement (39.7% vs. 27.7%). Steroid-free remission rates were comparable, but vedolizumab-treated patients experienced fewer overall and serious infections, reinforcing its safety profile [43] (Table 3).

Vedolizumab is also effective in CD. In the GEMINI 2 trial, vedolizumab significantly increased clinical remission compared with placebo at both week 6 (14.5% vs. 6.8%; *p* = 0.02) and week 52 (39% vs. 21.6%; *p* < 0.001) [35]. In the GEMINI 3 trial, in TNF-experienced CD patients, vedolizumab did not meet remission endpoint at week 6, but showed significantly greater remission rates at week 10 compared with placebo (26.6% vs. 12.1%; *p* = 0.001) [65]. Real-world studies have corroborated vedolizumab’s effectiveness in CD. Although no head-to-head trials in CD are available, retrospective analyses suggest vedolizumab offers durable efficacy and steroid-sparing benefits comparable to other advanced therapies, particularly anti-TNF such as infliximab, adalimumab and certolizumab, with a potential safety advantage due to its gut-selectivity [66]. Since its FDA approval for UC and for CD, subcutaneous vedolizumab has been validated as an effective maintenance therapy. In VISIBLE 1 trial, clinical remission at 52 weeks was 46.2% with SC vedolizumab versus 14.3% with placebo (*p* < 0.001) in UC patients [67]. In VISIBLE 2 trial subcutaneous vedolizumab induced higher remission at week 52 compared with placebo in CD patients (48% vs. 34.3%; *p* = 0.008) [68].

Natalizumab, another humanized monoclonal antibody, targets both α4β7 (blocking MAdCAM-1) and α4β1 integrin (blocking vascular cell adhesion molecule-1, VCAM-1), the latter being expressed in the gut as well as in the central nervous system. This broader mechanism increases the risk of progressive multifocal leukoencephalopathy (PML) due to JC virus reactivation [60,69]. Natalizumab’s efficacy in CD was established in the ENACT and ENCORE clinical trials. In ENACT-1, response rates at week 10 were higher with Natalizumab versus placebo (56% vs. 49%, *p* = 0.05); in ENACT-2, responders showed significantly higher maintenance of response (61% vs. 28%, *p* < 0.001), and remission (44% vs. 26%, *p* < 0.001) [36]. In ENCORE, among patients with active inflammation, natalizumab achieved response in 48% vs. 32% (*p* = 0.002) and remission in 26% vs. 16% (*p* = 0.04) compared with placebo [70]. Approved for relapsing multiple sclerosis in 2004 and for moderate-to-severe CD in 2008 by the FDA [71], natalizumab is reserved for selected IBD patients due to the rare but serious risk of PML, and it is not currently available in Europe for IBD treatment.

### 4.2. S1PR Modulators

The S1PR modulators are oral small molecules that inhibit immune cell trafficking and are currently approved for the treatment of moderate-to-severe UC. The two approved agents are ozanimod and the more recently authorized etrasimod [72].

Ozanimod selectively targets S1P1 and S1P5 receptors. These receptors are differentially expressed in various tissues; S1P1 is predominantly found in the vascular endothelium and lymphocytes, S1P3 is highly expressed in the heart and lungs, while S1P5 is mainly expressed in the central nervous system. This receptor selectivity is clinically relevant, particularly in minimizing cardiovascular (CV) risk. Non-selective S1PR modulators or those with S1P3 affinity may induce bradycardia or atrioventricular block due to negative chronotropic effects. Although S1P1 modulation can reduce heart rate, this effect is transient and effectively managed with a 7-day dose titration. Ozanimod, by avoiding S1P3 modulation, offers a favorable CV safety profile, making it suitable even for patients with low-to-moderate CV risk, following appropriate baseline assessment. However, due to its affinity for S1P1 receptors, which are also expressed in the eye, ozanimod may cause macular edema, particularly in high-risk individuals (e.g., with diabetes, uveitis or prior macular edema), for whom fundoscopic examination is advised before initiating treatment [73].

The phase 3 TRUE NORTH trial [17] demonstrated significant efficacy for ozanimod in both induction and maintenance phases in UC. At week 10, clinical remission, clinical response and mucosal healing were significantly higher with ozanimod than placebo (18.4% vs. 6%, 47.8% vs. 25.9%, and 12.6% vs. 3.7%, respectively). These effects were sustained through week 52, with 37% of responders remaining in remission compared with 18.5% with placebo. Ozanimod was well tolerated, with low rates of serious infections and no new safety signals. Based on these results, ozanimod received FDA and EMA approval in 2021 for moderate-to-severe UC [74].

Long-term data from the TOUCHSTONE open-label extension phase 2 study [75] confirmed durable efficacy of ozanimod for up to 200 weeks, with clinical response and remission rates of 93.3% and 82.7%, respectively. Endoscopic improvement and histologic remission were maintained from week 56 through week 104. Subgroup analyses showed similar outcomes in anti-TNF-naïve and -experienced patients.

Real-world evidence confirmed ozanimod’s effectiveness in treatment-refractory UC. A prospective-1-year observational study in 45 patients (82% previously exposed to biologics or small molecules) reported clinical response and remission rates of 58% and 53% at week 10, though these declined to 25% and 29% by week 52. No significant efficacy differences were observed between therapy-naïve and previously treated patients [76]. In Crohn’s disease, preliminary data from the YELLOWSTONE trial showed encouraging efficacy results and a favorable safety profile for ozanimod in patients with moderate to severe disease, suggesting that it may eventually lead to approval his indication [77].

Etrasimod is a second-generation S1PR modulator with high selectivity for S1P1, S1P4 and S1P5, minimal activity on S1PR3, and none on S1PR2. As with ozanimod, interaction with S1PR1 may affect cardiac rhythm and ocular tissues, thus, ECG and fundoscopic examination are recommended prior to treatment initiation in all patients [74,78]. The ELEVATE UC-12 and-52 phase 3 trials showed significant benefit for etrasimod. In the 12-week induction trial, clinical remission was achieved in 25% vs. 15% with placebo (*p* = 0.026). In the treatment-through 52-week trial, remission rates were 27% vs. 7% at week 12, and 32% versus 7% at week 52 (*p* < 0.0001). Clinical response and endoscopic improvement were also superior with etrasimod [18]. Based on these results, etrasimod was approved by the FDA and EMA in 2023 for moderate-to-severe UC [78]. To date, no real-world data are available.

## 5. Janus Kinase Inhibitors

Janus Kinases (JAKs) are cytoplasmic tyrosine kinases that mediate intracellular signalling of cytokines by associating with the intracellular domains of cytokine receptors. Upon cytokine binding to its membrane receptor, JAKs become activated and phosphorylate members of the signal transducer and activator of transcription (STAT) family inside the cells. Phosphorylated STAT dimerizes and translocates to the nucleus, where they bind specific DNA-regulatory elements to modulate transcription of target genes [79,80]. Through this mechanism, the JAK-STAT pathway transduces extracellular cytokine signals into nuclear transcriptional responses, influencing key biological processes, including apoptosis, proliferation, migration, development, and differentiation of various cell types (T cells, B cells, NK cells, macrophages and epithelial cells) [81] (Figure 1). More than 50 cytokines (including interferons, interleukins, and growth factors) are now recognized to signal through the JAK-STAT pathway, regulating cell differentiation, metabolism, homeostasis and immune-response.

Unlike monoclonal antibodies used in IBD treatment, which selectively target a single cytokine (e.g., TNFα, and IL-23), JAK inhibitors (JAKi) simultaneously modulate multiple inflammatory pathways. This broad activity may provide therapeutic advantages but also carries a potentially greater risk of toxicity and adverse events [80]. Common non-serious adverse effects of JAKi include bone marrow suppression (anaemia and pancytopenia), hepatotoxicity (elevated transaminases and bilirubin), and dyslipidaemia, often requiring lipid-lowering therapy. JAKi are also associated with an increased incidence of infections, particularly Herpes Zoster [82].

The JAK family comprises four kinases: JAK1, JAK2, and TYK2, which are broadly involved in immune system development and regulation, and JAK3, which is expressed predominantly in the hematopoietic system, especially in myeloid and lymphoid cells [79,80,81]. JAKi act by preventing JAK phosphorylation, thereby blocking STAT activation. As “small molecules”, they have substantially lower molecular weight than monoclonal antibodies with subsequent lower immunogenicity, are orally administered, and have a much shorter half-life (a few days versus weeks to months) [53].

The first JAKi developed for the treatment of IBD was tofacitinib, a first-generation pan-JAK inhibitor. It is approved for the treatment of moderate-to-severe UC based on phase 3 induction and maintenance trials. In the OCTAVE induction trial, remission at week 8 was achieved in 18.5% of patients versus 8.2% with placebo (*p* = 0.007). In the OCTAVE sustain trial, remission rates at week 52 were 40.6% with tofacitinib and 11.1% with placebo (*p* < 0.001) [19]. Although effective in UC, trials in CD failed to demonstrate therapeutic benefit [83]. As a pan-JAK inhibitor, tofacitinib blocks multiple JAK isoforms, resulting in broad modulation of inflammatory pathways but also in adverse effects, including cardiovascular events, thromboembolic complications such as pulmonary embolism and deep vein thrombosis, as well as a slight increase in malignancy risk. In rheumatoid arthritis a randomized trial reported a 4-year major adverse cardiovascular events (MACE) of 3.4% with tofacitinib 5 mg BID and 4.2% with 10 mg BID, compared with 2.5% with anti-TNF therapy, with higher risk in patients ≥ 65 years or with prior atherosclerotic cardiovascular disease [84]. While similar findings have not been observed in IBD, the EMA and FDA recommend JAKi be reserved for patients under 65 years of age without cardiovascular risk factors. Notably real-world studies and meta-analyses in rheumatoid arthritis have not shown significant increase in MACE compared with disease modifying antirheumatic drugs (DMARDs) or anti-TNF agents [85].

Given that JAK1 is thought to play a more prominent role than other JAK isoforms in driving intestinal inflammation, the development of highly selective JAK-1 inhibitors has been pursued to optimize efficacy while minimizing off target toxicity [80].

The two JAK 1 selective inhibitors approved for IBD are upadacitinib and filgotinib. JAK-1 selectivity is dose dependent, and at higher exposures both agents exert broader inhibition across the JAK family [86].

Upadacitinib is approved for both CD and UC. In U-EXCEL phase 3 induction [37] trial for CD, at week 12 clinical remission was achieved in 49.5% versus 29.1% with placebo, with endoscopic response rates of 45.5% versus 13.1% (*p* < 0.001). In the maintenance U-ENDURE trial [37], at week 52, clinical remission and endoscopic response rates with the 30 mg maintenance dose were 47.6% and 40.1%, respectively, compared with 15.1% and 7.3% for placebo; the 15 mg dose achieved lower but still statistically significant rates (*p* < 0.001 for all comparisons). In UC, the phase 3 U-ACHIEVE and U-ACCOMPLISH induction trials reported clinical remission at week 8 in 26% and 33% of patients versus 5% and 4% with placebo (*p* < 0.0001). In the U-ACHIEVE maintenance trial, week 52 remission occurred in 42% of patients receiving 15 mg and in 52% of those receiving 30 mg, compared with 12% with placebo (*p* < 0.0001) [20].

Filgotinib is approved for UC, based on data from SELECTION induction phase 3 trials, showing clinical remission rates of 26.1% versus 15.3% with placebo at week 10 (*p* = 0.0157), and from SELECTION maintenance phase 3 trial in which the proportion of patients in clinical remission was 37.2% versus 11.2% with placebo at week 58 (*p* < 0.0001) [21]. Although not approved for CD, phase 2 and phase 3 trials demonstrated its efficacy, particularly in maintaining disease remission, with week 58 remission rates of 43.8% versus 26.4%, (*p* = 0.0382) and endoscopic remission rates of 30.4% versus 9.4% (*p* = 0.0038). Data from rheumatoid arthritis suggest that filgotinib has the most favorable cardiovascular safety profile among JAKi, with no appreciable increase in MACE rates [85].

## 6. The Role of Microbiome: Probiotics and Postbiotics

In IBD, alterations in the total number, diversity, and richness of microbial communities (collectively referred to as dysbiosis) can actively contribute to intestinal inflammation by disrupting mucosal immune homeostasis [87]. Under physiological conditions, the healthy gut maintains a mutualistic host–microbe equilibrium through several immune mechanisms, including intestinal secretory IgA, which plays a central role in preserving symbiosis and preventing the overgrowth of pathogenic species [88]. These insights have raised interest in microbiome-targeted interventions as potential therapeutic strategies. The mechanisms of action of microbiome-based therapies are heterogeneous and depend on strain, dose, and host factors. Their effects range from direct antibacterial activity (e.g., metabolite production) to non-immunological functions such as nutrient competition, mucus production, pH modification, reinforcement of tight junctions, and promotion of tissue repair. Unlike advanced therapies that primarily modulate inflammatory pathways, probiotics and postbiotics aim to address the underlying dysbiosis and epithelial barrier dysfunction. By acting at the level of the gut lumen and mucosal surface, they could complement immune-targeted drugs, potentially enhancing mucosal healing and prolonging remission [89]. Probiotics have been shown to restore microbial balance and strengthen the intestinal barrier. Multi-strain formulations, in particular, have demonstrated some benefits in UC, improving barrier integrity, modulating immune pathways, and enhancing clinical response when used alongside advanced therapies [89]. Postbiotics—defined as microbial products such as metabolites, peptides, or inactivated bacterial components—represent a further evolution of this concept. By delivering defined microbial products (e.g., butyrate), postbiotics may promote epithelial integrity and regulatory immune responses, offering theoretical advantages in immunocompromised patients due to their favourable safety profile [87,89]. Importantly, because they are non-viable, they circumvent the rare but documented risk of probiotic-related infections in high-risk hosts. Despite these promising mechanistic insights, clinical evidence for probiotics and postbiotics in IBD remains limited. Results are highly strain-dependent, with inconsistent outcomes in Crohn’s disease and more encouraging, though not definitive, findings in UC [2,89]. Recent meta-analyses emphasize the need for more rigorous strain selection, optimized dosing strategies, and a better understanding of underlying mechanisms [90].

## 7. Future Perspectives

### 7.1. Advanced Combination Therapy

Despite the introduction of new therapies targeting different inflammatory pathways, a proportion of IBD patients still fail to achieve remission or experience secondary loss of response. Breaking the therapeutic ceiling remains a major challenge. Among the emerging approaches, advanced combination therapy (ACT), including biologic-biologic and biologic-small molecule pairings, represents a promising strategy. The immune dysregulation observed in IBD is a complex process involving a multifaceted interplay of cytokines, immune cells, and gut barrier dysfunction. Given that multiple inflammatory pathways are concurrently active in a single patient, a monotherapeutic approach targeting only one pathway may be insufficient to achieve adequate control of the inflammation [91].

The rationale for using advanced combination therapy is based on several key principles. The primary aim is to exploit pharmacodynamic complementarity by simultaneously targeting non-overlapping immune pathways to achieve greater disease control than either agent alone, thus avoiding potential mechanistic escape and loss of response [92]. Furthermore, combination therapy can offer a key “temporal advantage”. This involves using a fast-onset agent (e.g., JAKi) to induce control while a slower-onset maintenance agent (e.g., Vedolizumab, Ustekinumab) takes effect, allowing for a subsequent sequential de-escalation of the initial agent. Another advantage is the ability to address different disease manifestations. This is achieved by associating a gut-selective drug to target luminal inflammation with a systemic agent that can also address extraintestinal manifestations (EIMs) or concomitant immune-mediated inflammatory diseases (IMIDs).

The synergistic potential of combination therapy provides a strong rationale for its use. A compelling example is the association between anti-TNF and anti-IL-23 therapies. In patients refractory to anti-TNF treatment, there is a notable upregulation of mucosal IL-23p19, IL-23R and IL-17A. This is accompanied by an expansion of apoptosis-resistant TNFR2+IL23R+ T cells, which are activated by IL-23 originating from CD14+ macrophages, a cell population more prevalent in non-responders. As functional studies have demonstrated, IL-23 is able to counteract the pro-apoptotic effects of anti-TNF agents on mucosal T cells. This mechanistic insight suggests that targeting the IL-23 pathway can overcome resistance to anti-TNF therapy, leading to a synergistic therapeutic outcome [55].

Another example of combined therapeutic effect is the association of an agent that targets leukocyte trafficking with a cytokine pathway blocker. Specifically, while the α4β7 integrin inhibitor vedolizumab effectively reduces gut homing of immune cells, it does not inhibit the inflammatory activity of T-cells that have already infiltrated the mucosa. When combined with a cytokine inhibitor (such as ustekinumab or an anti-TNF agent), this approach provides a complementary benefit by suppressing local effector activity, thereby leading to improved therapeutic outcomes [93].

ACT may be considered in two main scenarios: (i) patients with difficult-to-treat IBD, often after multiple biologic failures, or when surgery is not an option; and (ii) patients with concomitant active extraintestinal manifestations or other immune-mediated diseases.

The most frequently reported combination is vedolizumab plus an anti TNF-α, which exploits the gut-selectivity effect of vedolizumab to reduce systemic adverse events. In a systematic analysis of 279 patients, Ahmed et al. [94] reported vedolizumab + anti TNF-α as the most common pairing (48%), followed by vedolizumab + ustekinumab (19%), anti TNF-α + ustekinumab (7%), and tofacitinib + vedolizumab (≈20%). Most patients had CD with multiple prior biologic failures, and pooled clinical and endoscopic remission rates were 58.8% (95% CI, 42–74.5%) and 34.3% (95% CI, 23.5–46.1%), respectively. Similarly, a systematic review and meta-analysis by Alayo, including 273 IBD patients [95], confirmed vedolizumab + anti-TNF-α as the most frequent pairing among biologics, while vedolizumab + tofacitinib was the most common biologic-small molecule combination. Reported remission rates ranged from 47% to 55% for clinical endpoints and 18% to 25% for endoscopic endpoints, depending on the regimen.

Data on JAKi in combination are expanding. Bhaskar et al. [96] evaluated 79 patients with CD or UC, of whom 90.7% received a JAKi (tofacitinib or upadacitinib) plus a biologic, demonstrating significant clinical and endoscopic improvements. The most frequent adverse events were upper respiratory infections (21.6%) and dermatological manifestations (8.2%). Other real-world reports and meta-analyses confirm infections as the main safety concern, although current evidence is limited by small cohorts [96,97]. Evidence from randomized controlled trials is still limited [98]. The VEGA trial [99], a controlled proof-of-concept study, showed that guselkumab plus golimumab induced higher remission rates in UC (36.6%) compared with either monotherapy (21.1%).

More recently, EXPLORER trial [100] further explored triple combination therapy (vedolizumab, adalimumab, methotrexate) in 55 biologic-naïve but difficult-to-treat CD patients, achieving clinical and endoscopic remission rates of 55% and 35%, respectively. However, the absence of a comparator arm was a major limitation, and post hoc Bayesian analysis was required to contextualize outcomes, comparing the results with estimated remission rates from biological monotherapy and placebo.

The efficacy of advanced combination therapies is being evaluated in several ongoing studies, including the VICTRIVA (NCT06227910) trial, which is evaluating the efficacy and safety of vedolizumab plus upadacitinib in the induction phase for Crohn’s disease. In addition, the DUET CD (NCT05242471) and DUET UC (NCT05242484) trials are Phase 2b randomized, double-blind, placebo-controlled studies that are evaluating the efficacy and safety of golimumab and guselkumab in varying doses for both induction and maintenance therapy.

Beyond intestinal disease control, ACT has shown promising results in managing EIMs and IMIDs and the choice of combination can be tailored to the specific manifestations. For rheumatological manifestations like ankylosing spondylarthritis and rheumatoid arthritis, adding an anti-TNF or a JAK inhibitor can be considered, especially with vedolizumab, for a gut-selective treatment that reduces the risk of adverse events.

A European retrospective multicentre study [101] found that 33 out of 104 dual-combination regimens were used specifically for EIMs/IMIDs, with anti-integrin + anti-IL and anti-TNF + anti-integrin combinations being the most common. Overall efficacy reached 81% for conditions such as ankylosing spondylarthritis, psoriasis, psoriatic arthritis, and rheumatoid arthritis. In psoriatic diseases, anti-TNF, JAKi, anti-IL-23, and anti-IL-12/23 agents have been successfully combined, while in hidradenitis suppurativa anti-TNF and JAKi, particularly upadacitinib, have shown efficacy [92].

Safety remains the main concern. 

While this approach may offer greater efficacy, it also carries the potential for increased adverse events. To mitigate this risk, a common strategy is to pair an agent with a favourable safety profile (e.g., vedolizumab or anti IL-12/23 or anti IL-23) with a second agent that has a greater risk of adverse events (e.g., an anti-TNF or JAK inhibitor). According to data from a European cohort, approximately 10% of patients experienced opportunistic or hospitalized infections, corresponding to an infection rate of 8.2 per 100 patient-years [101]. Most patients who developed a severe infection had an associated EIM or IMID, with 60% of these individuals also receiving a concomitant steroid and/or immunomodulator [101]. In contrast, results from meta-analyses suggest a higher pooled infection rate, reaching up to 20% [94]. Real-world data on JAK inhibitor plus biologic combinations also emphasize infections as a key concern, highlighting the need for larger comparative safety studies due to small sample sizes. Data on the long-term safety and the risk of malignancies are still lacking.

The selection of ACT is currently guided by clinical factors such as disease activity, phenotype, prior treatment response, comorbidities, and the presence of EIMs. While the choice of dual therapy is more straightforward for managing concomitant EIMs, decision-making is more challenging for patients with refractory intestinal disease. A major limitation is the lack of studies comparing the efficacy of different dual therapies, which is compounded by the unavailability of reliable biomarkers to predict treatment response. Ultimately, more data are needed to fully define its role, to determine whether true synergistic effects can be achieved, and to enable the selection of the optimal combination for each patient.

### 7.2. Emerging Biomarkers for the Selection of Personalized Therapies: Microbiome Signatures, Proteomic and Organoids

Considering the growing number of effective therapies for IBD, there is a strong clinical need for biomarkers capable of guiding the selection of the most appropriate strategy for individual patients. Several promising approaches are currently under investigation, including the analysis of microbiome signatures, proteomic and transcriptomic profiling, and the use of patient-derived organoids. Alterations in the gut microbiome, with reduced bacterial diversity, expansion of potentially harmful bacteria such as Enterobacteriaceae and Fusobacteriaceae [102], and depletion of beneficial species such as Faecalibacterium prausnitzii [103] and other butyrate-producing bacteria [104], have been consistently described in IBD. This dysbiosis contributes to mucosal inflammation and disease severity, and recent studies suggest that specific microbial patterns may correlate with disease activity and therapeutic response. For instance, a prospective cohort study identified key bacterial species able to predict mesalamine treatment failure in UC patients [105]. Distinct fecal microbiome profiles have also been described in Chinese patients with Crohn’s disease responsive or nonresponsive to ADA [106], and baseline bacterial and fungal signatures have been associated with IFX responsiveness in both UC and CD [107]. These observations suggest that faecal microbiome signatures may become useful biomarkers for predicting therapeutic outcomes, although larger prospective studies are needed to validate their utility and to assess their applicability to newer advanced therapies, including small molecules. Notably, a prospective longitudinal observational study, currently ongoing in Canada, is exploring microbiome profiles in CD patients treated with advanced therapies, with the aim of developing predictive models of response [108].

In parallel, proteomics and transcriptomics are being explored as tools to refine therapeutic decisions. Proteomics, which investigates the entire set of proteins expressed by cells and tissues, and transcriptomics, which focuses on the global RNA profile, have both been evaluated in IBD. A proteomic study in paediatric patients with moderate-to-severe CD demonstrated that baseline profiles of infliximab-modulated inflammatory proteins could predict clinical response to both conventional and infliximab-based therapies [109,110]. Other studies combining immunophenotypic and transcriptomic characterization of peripheral and tissue immune cells have reported the potential to predict vedolizumab response in both UC and CD [111]. Interestingly, in this study transcriptomic data were integrated with clinical parameters and biomarkers in a machine learning model, which improved the prediction of response. These findings suggest that future applications of artificial intelligence integrating clinical, genomic, transcriptomic, proteomic, and microbiomic data could substantially advance precision medicine in IBD [112].

Another promising avenue is represented by intestinal organoids. These three-dimensional, lab-grown epithelial structures maintain the genetic, epigenetic, and functional properties of the original tissue for months in vitro [113]. Organoids can be derived directly from patient stem cells, enabling in vitro testing of growth factors, cytokines, and drugs, such as IFX [114] and tofacitinib [115], under personalized conditions. Beyond their utility in drug selection, organoids hold potential as regenerative tools, with experimental studies suggesting their transplantation into damaged intestinal segments as a novel therapeutic strategy [116]. Nevertheless, important challenges remain regarding the reproducibility, standardization, and reliability of organoid-based approaches before their translation into routine clinical practice [117].

## 8. Conclusions

The treatment of IBD remains a formidable challenge, driven by the simultaneous activation of multiple inflammatory pathways, the frequent occurrence of EIMs/IMIDs, and the high rate of therapeutic loss of response. While substantial progress has been made with currently available therapies and encouraging results are emerging from studies on ACT and novel agents, breaking the current therapeutic ceiling will require further advances. Innovative approaches—including the exploration of microbiota-targeted strategies, proteomic and transcriptomic profiling, and organoid-based technologies—hold promise, but continued research is essential to translate these insights into truly personalized and more effective care. In this context, the integration of conventional therapies with advanced agents and microbiome-based strategies may represent a promising path toward more effective and durable disease control.

## Figures and Tables

**Table 1:** Representative outcomes from clinical trials of biologics and small molecules in Ulcerative Colitis.

Agent	Clinical Trial	N° of Patients	Study Period	Outcomes
**Infliximab**	ACT1 [6]	Placebo (n = 121) IFX 5 mg/kg (n = 121) IFX 10 mg/kg (n = 122)	8 weeks	Clinical response: 69% IFX 5 mg/kg vs. 61% IFX 10 mg/kg vs. 29% placebo Mucosal healing: 62% IFX 5 mg/kg vs. 60% IFX 10 mg/kg vs. 33% placebo
**Adalimumab**	ULTRA-I [7]	Placebo (n = 130) ADA 160/80 mg (n = 130)	8 weeks	Clinical remission: 18% ADA 160/80 mg vs. 9% placebo
	ULTRA-II [8]	Placebo (n = 260) ADA 40 mg q2w (n = 258)	52 weeks	Clinical remission: 17% ADA 160/80 vs. 8% placebo
**Golimumab**	PURSUIT-SC [9]	Placebo (n = 251) GOL 200/100 mg (n = 253)	6 weeks	Clinical remission: 18% GOL 200/100 mg vs. 6% placebo
	PURSUIT-M [10]	Placebo (n = 154) GOL 100 mg q4w (n = 151)	54 weeks	Maintained clinical response: 50% GOL 100 mg q4w vs. 23% placebo
**Ustekinumab**	UNIFI [11]	Placebo (n = 319) UST 130 mg (n = 320) UST 6 mg/kg (n = 322)	8 weeks	Clinical remission: 16% UST 130 mg vs. 15.5% UST 6 mg/kg vs. 5% placebo
**Risankizumab**	INSPIRE [12]	Placebo (n = 325) RIS 1200 mg (n = 652)	12 weeks	Clinical remission: 20% RIS 1200 mg vs. 6% placebo
	COMMAND [13]	Placebo (n = 183) RIS 180 mg (n = 179)	52 weeks	Clinical remission: 40% RIS 180 mg vs. 25% placebo
**Mirikizumab**	LUCENT-1 [14]	Placebo (n = 294) MIRI 300 mg (n = 868)	12 weeks	Clinical remission: 24% MIRI 300 mg vs. 13% placebo
	LUCENT-2 [14]	Placebo (n = 179) MIRI 200 mg (n = 365)	40 weeks	Clinical remission: 50% MIRI 200 mg vs. 25% placebo
**Guselkumab**	QUASAR Induction [15]	Placebo (n = 280) GUS 200 mg (n = 421)	12 weeks	Clinical remission: 23% GUS 200 mg vs. 8% placebo
	QUASAR maintenance [15]	Placebo (n = 190) GUS 200 mg e4w (n = 190) GUS 100 mg e8w (n = 188)	44 weeks	Clinical remission: 50% GUS 200 mg e4w vs. 45% GUS 100 mg e8w vs. 19% placebo
**Vedolizumab**	GEMINI 1 [16]	Placebo (n = 161) VEDO 300 mg (n = 374)	6 weeks	Clinical response: 47% VEDO 300 mg vs. 25% placebo
**Ozanimod**	TRUE NORTH [17]	Placebo (n = 216) OZA 0.92 mg (n = 429)	10 weeks	Clinical remission: 18% OZA 0.92 mg vs. 6% placebo
**Etrasimod**	ELEVATE UC-12 [18]	Placebo (n = 116) ETR 2 mg (n = 238)	12 weeks	Clinical remission: 25% ETR vs. 15% placebo
**Tofacitinib**	OCTAVE Induction [19]	Placebo (n = 138) TOFA 10 mg BID (n = 476)	8 weeks	Clinical remission: 18.5% TOFA 10 mg BID vs. 8% placebo
	OCTAVE Sustain [19]	Placebo (n = 198) TOFA 10 mg BID (n = 197)	52 weeks	Clinical remission: 41% TOFA 10 mg vs. 11% placebo
**Upadacitinib**	U-ACHIEVE Induction [20]	Placebo (n = 238) UPA45 mg (n = 236)	8 weeks	Clinical remission: 25% UPA 45 mg vs. 5% placebo
	U-ACHIEVE maintenance [20]	Placebo (n = 223) UPA 15 mg (n = 225) UPA 30 mg (n = 233)	52 weeks	Clinical remission: 42% UPA 15 mg vs. 52% UPA 30 mg vs. 12% placebo
**Filgotinib**	SELECTION A [21]	Placebo (n = 137) FIL 200 mg (n = 245)	10 weeks	Clinical remission: 26% FIL 200 mg vs. 15% placebo
	SELECTION B [21]	Placebo (n = 142) FIL 200 mg (n = 262)	10 weeks	Clinical remission: 11.5% FIL 200 mg vs. 4% placebo
	SELECTION maintenance [21]	Placebo (n = 93) FIL 200 mg (n = 301)	58 weeks	Clinical remission: 37% FIL 200 mg vs. 11% placebo

**Table 2:** Representative outcomes from clinical trials of biologics and small molecules in Crohn’s Disease.

Agent	Clinical Trial	N of Patients	Study Period	Outcomes
**Infliximab**	ACCENT-1 [22]	Placebo (n = 110) IFX 5 mg/kg (n = 113) IFX 10 mg/kg (n = 112)	30 weeks	Clinical remission: 39% IFX 5 mg q8w vs. 45% IFX 10 mg q8w vs. 21% placebo
	SONIC [23]	AZA (n = 170) 30.0 IFX 5 mg/kg (n = 169) IFX+AZA (n = 169)	50 weeks	Clinical remission: IFX+AZA 57% vs. IFX 44% vs. AZA 30%
**Adalimumab**	CLASSIC-I [24]	Placebo (n = 74) ADA 40/20 mg (n = 75) ADA 80/40 mg (n = 75) ADA 160/80 mg (n = 76)	4 weeks	Clinical remission: 36% ADA 160/80 mg vs. 12% placebo vs. 24% ADA 80/40 mg vs. 18% 40/20 mg
	CLASSIC-II (maintenance) [25]	Placebo (n = 18) ADA 40 mg q2w (n = 19) 79/79 ADA 40 mg weekly (n = 18)	56 weeks	Clinical remission: 79% ADA q4w vs. 83% weekly vs. 44% placebo
	GAIN [26]	Placebo (n = 166) Ada 160/80 mg (n = 159)	4 weeks	Clinical remission: 21% ADA 160/80 mg vs. 7% placebo
	EXTEND [27]	ADA induction/placebo (n = 65) ADA 40 eow (n = 64)	52 weeks	Mucosal healing: 24% ADA vs. 0% placebo
**Certolizumab**	PRECiSE 1 [28]	Placebo (n = 331) CER 400 mg (n = 331)	4 weeks	Clinical response: 35% CER 400 mg vs. 27% placebo
	PRECiSE 2 [29]	Placebo (n = 331) CER 400 mg q4w (n = 331)	26 weeks	Clinical response: 48% CER 400 mg q4w vs. 29% placebo
**Ustekinumab**	UNITI [30]	Placebo (n = 210) UST 6 mg/kg (n = 209)	8 weeks	Clinical remission: 34% UST 6 mg/kg vs. 6.5% placebo
**Risankizumab**	ADVANCE [31]	Placebo (n = 175) RISA 600 mg iv (n = 336) RISA 1200 mg iv (n = 339)	12 weeks	Clinical remission: 45% RISA vs. 2% placebo
	MOTIVATE [31]	Placebo (n = 187) RISA 600 mg iv (n = 191) RISA 1200 mg iv (n = 191)		Clinical remission: 42% RISA vs. 19% placebo
	FORTIFY [32]	Placebo (n = 164) RISA 180 mg sc (n = 157) RISA 360 mg sc (n = 141	52 weeks	Clinical remission: 55% RISA 180 mg vs. 52% RISA 360 mg vs. 41% placebo
**Mirikizumab**	VIVID-1 [33]	Placebo (n = 199) MIRI 900/300 mg (n = 579) USTE 6 mg/kg-90 mg (n = 287)	52 weeks	Clinical remission: 45% MIRI vs. 20% placebo
**Guselkumab**	GRAVITI [34]	Placebo (n = 117) GUS 400/100 mg (n = 115) GUS 400/200 mg (n = 115)	12 weeks	Clinical remission: 56% GUS 400/200 mg vs. 54% GUS 400/100 vs. 21% placebo
**Vedolizumab**	GEMINI 2 [35]	Placebo (n = 148) VDZ 300 mg (n = 220)	6 weeks	Clinical remission: 14.5% VDZ 300 mg vs. 7% placebo
			52 weeks	Clinical remission: 39% VDX 300 mg vs. 22% placebo
**Natalizumab**	ENACT-1 (induction) [36]	Placebo (n = 181) NAT 300 mg (n = 724)	10 weeks	Clinical response: 56% NAT vs. 49% placebo
	ENACT-2 (maintenance) [36]	Placebo (n = 169) NAT 300 mg e4w (n = 170)	36 weeks	Clinical response: 61% NAT 300 mg vs. 28% placebo
**Upadacitinib**	U-EXCEL [37]	Placebo (n = 176) UPA 45 mg (n = 350)	12 weeks	Clinical remission: 49.5% UPA 45 mg vs. 29% placebo
	U-ENDURE [37]	Placebo (n = 165) UPA 30 mg (n = 168) UPA 15 mg (n = 169)	52 weeks	Clinical remission: 48% UPA 30 mg vs. 37% UPA 15 mg 15% placebo

**Table 3:** Summary of representative head-to-head clinical trials and their main outcomes.

Trial	Arms	N° of Patients	Study Period	Outcomes
**VARSITY [43]**	Vedolizumab vs. Adalimumab in moderate-to-severe UC	769 (383 VEDO, 386 ADA)	52 weeks	Clinical remission: 31% VEDO vs. 22.5% ADA Endoscopic improvement: 40% VEDO vs. 28% ADA
**SEAVUE [44]**	Adalimumab vs. Ustekinumab in moderate-to-severe CD	386 (195 ADA, 191 UST)	52 weeks	Clinical remission: 61% ADA vs. 65% UST Endoscopic remission: 35% ADA vs. 36% UST
**CYSIF [42]**	Infliximab vs. Cyclosporine in steroid-refractory ASUC	115 (58 cyclosporine, 57 IFX)	98 days	Colectomy rate: 41% IFX vs. 48% CYC (not statistically significant). No meaningful difference in quality of life.

## Data Availability

No new data were created or analyzed in this study.
